# Lean MASLD and IBD: Exploring the Intersection of Metabolic Dysfunction and the Gut–Liver Axis

**DOI:** 10.3390/life15020288

**Published:** 2025-02-12

**Authors:** Adrian Rotaru, Remus Stafie, Ermina Stratina, Sebastian Zenovia, Robert Nastasa, Horia Minea, Laura Huiban, Tudor Cuciureanu, Cristina Muzica, Stefan Chiriac, Irina Girleanu, Ana-Maria Singeap, Catalin Sfarti, Carol Stanciu, Anca Trifan

**Affiliations:** 1 Department of Gastroenterology, Grigore T. Popa University of Medicine and Pharmacy, 700115 Iasi, Romania; adrianrotaru94@yahoo.com (A.R.); stratina.ermina@yahoo.com (E.S.); sebastianzenovia20@gmail.com (S.Z.); robertnastasa948@gmail.com (R.N.); horia.minea@yahoo.com (H.M.); huiban.laura@yahoo.com (L.H.); drcuciureanutudor@gmail.com (T.C.); lungu.christina@yahoo.com (C.M.); gilda_iri25@yahoo.com (I.G.); anamaria.singeap@yahoo.com (A.-M.S.); cvsfarti@gmail.com (C.S.); stanciucarol@yahoo.com (C.S.); ancatrifan@yahoo.com (A.T.); 2 Institute of Gastroenterology and Hepatology, “St. Spiridon” University Hospital, 700111 Iasi, Romania

**Keywords:** MASLD, lean, IBD, CUN-BAE, gut–liver axis

## Abstract

Metabolic dysfunction-associated steatotic liver disease (MASLD) challenges traditional paradigms by manifesting in lean individuals. The link between MASLD and inflammatory bowel disease (IBD) underscores the importance of the gut–liver axis in disease progression and chronic inflammation. This study evaluates MASLD prevalence, clinical characteristics, and diagnostic predictors in lean individuals with and without IBD. This prospective study included 387 lean patients. Hepatic steatosis and fibrosis were assessed using vibration-controlled transient elastography (VCTE). Anthropometric, clinical and biological data were compared. The subgroup analyses focused on MASLD patients with and without IBD. MASLD was present in 34.1% of lean individuals and 46.3% of those who were lean with IBD. MASLD patients had increased visceral adiposity (CUN-BAE: 31.21 ± 5.42 vs. 24.57 ± 6.49, *p* < 0.001) and metabolic dysfunction, including dyslipidemia and elevated fasting glucose. IBD-MASLD patients exhibited greater hepatic steatosis and systemic inflammation. CUN-BAE outperformed FLI and HSI in predicting liver steatosis, especially in IBD patients (AUC = 0.806). Lean MASLD, particularly in IBD patients, highlights the need for tailored diagnostic and management strategies. The gut–liver axis plays a key role in disease progression, and the CUN-BAE index demonstrates superior accuracy for identifying liver steatosis.

## 1. Introduction

As the global rates of obesity and type 2 diabetes (T2D) rise, so does the prevalence of metabolic dysfunction-associated steatotic liver disease (MASLD). MASLD currently affects 38% of adults and 7–14% of children and adolescents, with the adult incidence estimated to exceed 55% by 2040. Moreover, MASLD has been thought to be the hepatic manifestation of the metabolic syndrome, and the more components of metabolic features present, the higher the risk for advanced fibrosis and mortality [1,2]. Of note is the fact that around 25% of the patients with MASLD will develop metabolic dysfunction-associated steatohepatitis, conditions that can lead to liver cirrhosis and hepatocellular carcinoma [3,4].

Although obesity is a well-recognized metabolic comorbidity linked to MASLD, some individuals develop the condition despite having a normal body mass index (BMI), a phenomenon known as lean MASLD. Despite their BMI falling within the normal range, these individuals may exhibit an abnormal distribution of body fat, which plays a critical role in the development of metabolic complications [5,6]. Abdominal fat is strongly associated with insulin resistance and serves as a significant source of free fatty acids, contributing to MASLD progression. Studies have shown that waist circumference is a stronger predictor of mortality in MASLD patients than BMI, leading many experts to recommend its use in risk assessment. Additionally, research indicates that individuals with lean MASLD can experience liver disease progression and outcomes comparable to those classified as non-lean [7,8].

MASLD has emerged after recent changes in the nomenclature of non-alcoholic fatty liver disease (NAFLD), which has been used for over two decades. These changes are the result of a longstanding apprehension regarding the use of terms such as “alcoholic” and “fatty” to describe the hepatic manifestation of a systemic metabolic disorder that is primarily characterized by cardiovascular outcomes. In this regard, the term “non-alcoholic” failed to accurately represent the disease’s etiology, which led to the need for a nomenclature change [9]. The term NAFLD has been replaced by MASLD, which falls within the broader category of steatotic liver disease (SLD). The decision was made to restrict the use of the new term to patients that have at least one of the five cardiometabolic risk factors that were previously recognized as fundamental characteristics of the metabolic syndrome. Individuals who do not have any metabolic risk factors and do not have any other known cause of steatotic liver disease would be categorized as having cryptogenic liver steatosis. Moreover, a new category, referred to as MetALD, has been established to specifically define individuals with MASLD who have a higher weekly alcohol consumption [10].

Lean MASLD, often characterized by a BMI of less than 25 kg/m^2^, challenges the conventional perception that fatty liver disease is primarily a consequence of obesity. Studies have shown that lean patients with MASLD exhibit a comparable incidence of cardiovascular events to their non-lean counterparts, despite typically having a lower prevalence of traditional metabolic risk factors such as diabetes, hypertension, and dyslipidemia [11,12,13]. This paradoxical finding underscores the necessity for heightened clinical awareness and targeted cardiovascular risk management in this subgroup.

As previously noted, lean patients with SLD represent a clinical and diagnosis challenge due to their normal BMI, as the condition is often characterized by abnormal fat distribution, mostly increased visceral adiposity [14]. BMI, a traditional marker of obesity, falls short in evaluating body fat composition and distribution. It does not distinguish between lean mass and fat mass or assess fat distribution, making it an inadequate tool for identifying metabolic risks in normal-weight individuals [15]. As such, alternative metrics are necessary, such as body fat percentage (BF%) and indices that provide a more accurate assessment of adiposity. The Clínica Universidad de Navarra-Body Adiposity Estimator (CUN-BAE) index has emerged as a reliable tool for estimating body fat percentage based on BMI, age and sex. Its formula incorporates multiple factors to provide a nuanced understanding of body composition [16].

Another key feature of MASLD is the association with inflammatory bowel disease (IBD). Emerging data clearly indicate a connection between IBD and the recently introduced term MASLD. In addition, although there has been a rise in the occurrence of obesity among patients with IBD, individuals who develop MASLD frequently tend to be categorized as lean [17,18,19]. The gut–liver axis plays a pivotal role in the interplay between IBD and MASLD. Disruption of the intestinal barrier in IBD can lead to increased intestinal permeability, allowing the translocation of bacteria and endotoxins into the portal circulation, which can subsequently trigger hepatic inflammation and contribute to the pathogenesis of MASLD. Moreover, the chronic systemic inflammation associated with IBD exacerbates metabolic disturbances, further linking these two conditions [18,19,20,21].

Lean patients with liver steatosis are a significant and much neglected group that is frequently linked to a negative outlook. It is crucial to comprehend the distinct phenotype and risk profiles of these patients to create efficient preventative and treatment methods. The main objective of our study was to evaluate the prevalence and risk factors of MASLD in lean individuals both with and without IBD. Moreover, we aimed to investigate the relationship between hepatic steatosis and IBD as an expression of gut–liver axis disruption.

## 2. Materials and Methods

### 2.1. Participants

This study is a prospective investigation conducted at a tertiary referral hospital located in northeastern Romania. The study population consisted of lean individuals who were consecutively enrolled. Patient data were collected and analyzed from December 2023 to July 2024. All patients had their demographic data, anthropometric measures, clinical examination, and personal medical history documented. Vibration-controlled transient elastography (VCTE) with controlled attenuation parameter (CAP) assessment was performed on all patients. The study included individuals who were 18 years of age or older. Patients who reported excessive alcohol consumption (defined as daily consumption of more than 20 g for women and 30 g for men) based on alcohol questionnaires, had a history of recent consumption of steatogenic medication (including corticosteroids) or those who had been diagnosed with human immunodeficiency virus (HIV) or chronic viral hepatitis B, C or D or any other secondary causes of chronic liver disease were not included in the study. For patients diagnosed with IBD, we collected data on the diagnosis date, IBD subtype, disease location, disease pattern (specifically for Crohn’s Disease), presence of perianal disease, extraintestinal manifestations, history of surgery and both current and past treatments. Patients with unreliable VCTE with CAP examinations were also excluded from the study. Blood samples were collected from all patients and the following tests were performed in our center: anti-HCV Ab, HBsAg, total cholesterol, low-density lipoprotein cholesterol (LDL-c), HDL-c, triglycerides, total bilirubin, gammaglutamyl transpeptidase (GGT), alanine and aspartate aminotransferase (ALT, AST), fasting plasma glucose, platelets count, serum urea, creatinine, C-reactive protein (CRP), alkaline phosphatase (ALP), international normalized ratio (INR), ferritin and HIV antibodies. The present study received approval from the Ethics Committee of our hospital and was conducted in accordance with the ethical guidelines outlined in the Declaration of Helsinki. All participants provided written informed consent.

### 2.2. Diagnostic Criteria and Definitions

Patients included in our study were considered to be normal weight or lean if their BMI was between 18.5 and 24.9 kg/m^2^. CUN-BAE was used for the estimation of body fat in our cohort. This tool uses BMI, sex and age to approximate the percentage of body fat, stratifying the individuals depending on sex as follows: normal body fatness if CUN-BAE is ≤20% in men and ≤30% in women; overweight if CUN-BAE is 20–25% in men and 30–35% in women; and obesity if CUN-BAE is >25% BF in men and >35% in women [16].

The diagnosis of SLD was confirmed when the CAP value exceeded 248 dB/m. MASLD was defined in patients with SLD and in the presence of at least one of the cardiometabolic criteria stipulated in the new nomenclature change, excluding a BMI of more than 25 kg/m^2^: waist circumference ≥94 cm in men and ≥80 cm in women; fasting serum glucose ≥100 mg/dL or 2 h post-load glucose levels ≥140 mg/dL or HbA1c ≥ 5.7% or type 2 diabetes or treatment for type 2 diabetes; blood pressure ≥ 130/85 mmHg or specific antihypertensive drug treatment; plasma triglycerides ≥150 mg/dL or lipid-lowering treatment; plasma HDL-cholesterol ≤ 40 mg/dL (males) and ≤50 mg/dL (females) or lipid-lowering treatment [10,22,23].

### 2.3. Liver Steatosis and Fibrosis Evaluation

Patients underwent liver fibrosis and steatosis evaluation using the FibroScan^®^ 520 Compact model (Echosens, Paris, France). A single operator, with experience of over 500 VCTE determinations, conducted the assessments. Patients fasted for at least four hours prior to the procedure and were placed in a supine position with their right arm maximally abducted, optimizing the intercostal window for scanning the liver’s right lobe. Most of the patients were examined with the M-probe (3.5 MHz transducer frequency) due to their constitutional type. A measurement was valid if ten acquisitions were completed with an interquartile range divided by the median (IQR/M) of ≤30%. The CAP values are expressed in decibels per meter (dB/m) and range from 100 to 400 dB/m. Liver steatosis was diagnosed when the CAP value exceeded 248 dB/m. For LSM, the thresholds for hepatic fibrosis were as follows: 5.6 kPa for mild fibrosis (F1), 8 kPa for significant fibrosis (F2), 9.6 kPa for advanced fibrosis (F3) and 13 kPa for cirrhosis (F4) [22,24].

In addition, we calculated the hepatic steatosis index (HSI) and fatty liver index (FLI) for each patient to evaluate the presence of hepatic steatosis. The hepatic steatosis index uses the following formula, 8 × (ALT/AST) + BMI + 2 (if type 2 diabetes) + 2 (if female), and a score of >36 predicts the presence of hepatic steatosis. The fatty liver index uses triglycerides, BMI, gammaglutamyltransferase (GGT) and waist circumference in its formula. An FLI value below 30 suggests a low probability of fatty liver, whereas an FLI value of 60 or higher signifies a high likelihood of its presence [25,26].

### 2.4. Statistical Analysis

Descriptive statistics were calculated for all variables using IBM SPSS, Version 22.0 (IBM SPSS Inc., Chicago, IL, USA). The Kolmogorov–Smirnov test was employed to assess the normality of the distribution of numerical data. Continuous variables are typically represented using measures of central tendency, such as means, and measures of dispersion, such as standard deviation. On the other hand, categorical variables are often stated using numerical counts or proportions, commonly presented as percentages. The data were subjected to statistical analysis using various tests depending on the type of data and the distribution. For normally distributed data, an unpaired *t*-test was employed to compare continuous variables between groups. For categorical data, either a chi-squared test or Fisher’s exact test was used. In cases where the data were skewed, a Mann–Whitney U test or Kruskal–Wallis analysis of variance (ANOVA) test was utilized for comparison. The Pearson correlation coefficient (r) was employed to determine the relationship between two variables. A *p*-value less than 0.05 in a two-tailed test was deemed to be statistically significant. The analysis was conducted solely on datasets that were full. The receiver operating characteristics (ROC) curve was analyzed, and the area under the curve (AUROC) was calculated to establish diagnostic accuracy for noninvasive diagnosis tools.

## 3. Results

### 3.1. Overview of Study Cohort

In the final analysis, 387 lean patients were included. After a close evaluation, a distinct subgroup of 82 patients emerged, namely individuals with IBD, representing 21.2% of the cohort. Concerning age distribution, the mean age of the overall cohort was 50.9 ± 15.52 years, with IBD patients being significantly younger when compared to their non-IBD counterparts, as presented in Table 1. Among the overall cohort, the prevalence of MASLD was 34.1%. A significantly higher proportion of IBD patients (46.3%) had SLD compared to non-IBD patients (30.9%, *p* = 0.013). Moreover, the mean CAP value was higher in IBD patients compared to non-IBD patients, although the difference was not statistically significant (*p* = 0.067), with LSM values following the same trend.

Regarding lipid metabolism, IBD patients had significantly higher total cholesterol levels compared to non-IBD patients (*p* < 0.001). Similarly, triglyceride levels (*p* = 0.006), fasting plasma glucose (*p* = 0.014) and CRP levels (*p* = 0.033) were elevated in the IBD group. Of note is the fact that there were no significant differences in the prevalence of hypertension or type 2 diabetes mellitus between the two groups.

### 3.2. Comparison of MASLD vs. Non-MASLD Lean Patients

Lean patients with MASLD exhibited major differences concerning clinical and biological parameters when compared to their non-MASLD counterparts, as seen in Table 2. Of note is the fact that LSM values were significantly higher in the MASLD cohort (6.93 ± 2.39 kPa) compared to non-MASLD patients (5.86 ± 3.01 kPa, *p* < 0.001). Moreover, a significantly higher proportion of SLD patients had at least significant fibrosis (27.2%) compared to non-SLD patients (18.1%, *p* < 0.001).

MASLD patients had significantly higher total cholesterol levels (*p* = 0.018), LDL cholesterol levels (*p* < 0.001), triglyceride levels (*p* < 0.001) and fasting plasma glucose (*p* = 0.014). In contrast, HDL cholesterol levels were lower in the MASLD group (46.42 ± 12.82 mg/dL vs. 49.98 ± 13.65 mg/dL, *p* = 0.012).

Significant differences were noted between the two groups when the smoking status and comorbidities were evaluated. The proportion of active smokers (*p* = 0.014) and previous smokers (*p* = 0.094) was more important in the MASLD cohort. Moreover, hypertension was more prevalent in MASLD patients (*p* < 0.001), as was type 2 diabetes mellitus (*p* < 0.001). Lipid-lowering treatment was significantly more common among MASLD patients (25.7% vs. 7.45%, *p* < 0.001).

The HSI was significantly higher in MASLD patients (33.76 ± 4.39) compared to non-SLD individuals (31.15 ± 3.90, *p* < 0.001), reinforcing its utility in identifying steatosis. The same changes were observed regarding the FLI and CUN-BAE, with significantly higher values in the MASLD cohort.

Concerning the presence of cardiometabolic risk factors stipulated by the Delphi consensus in MASLD patients, in our cohort, 31 patients (23.6%) had one risk factor, 58 patients (43.8%) had two risk factors, 26 patients (19.6%) had three risk factors, 12 patients (9.3%) had four, and 5 patients (3.7%) had five cardiometabolic risk factors.

### 3.3. Comparison of Clinical Profiles Between IBD and Non-IBD MASLD Patients

A deeper examination of clinical characteristics between MASLD patients with and without IBD reveals a series of differences. IBD patients with MASLD tended to be younger than their non-IBD counterparts (*p* = 0.008). Height and weight were also significantly lower in IBD-MASLD patients, but despite their lower anthropometric measures, IBD-MASLD patients had slightly higher waist circumferences compared to non-IBD individuals (*p* = 0.038) (Table 3).

Concerning liver steatosis, IBD-MASLD patients presented higher CAP values than non-IBD MASLD individuals (*p* = 0.049). On the other hand, LSM values did not significantly differ between the subgroups (*p* = 0.977), with IBD and MASLD patients showing a slightly higher prevalence of at least significant fibrosis. Despite a higher BMI (*p* = 0.021), IBD-MASLD patients exhibited similar levels of fasting plasma glucose and lipid profiles compared to their non-IBD counterparts. Patients with MASLD and IBD had significantly lower AST levels (*p* = 0.003) and GGT levels (*p* = 0.001) compared to the MASLD-without-IBD cohort.

There were no significant differences in the proportions of active smokers (28.9% vs. 32.9%, *p* = 0.135) or previous smokers (18.4% vs. 20.2%, *p* = 0.836). The prevalence of hypertension (42.1% vs. 38.4%, *p* = 0.724) and type 2 diabetes mellitus (23.6% vs. 22.3%, *p* = 0.892) was similar between the two groups. Although lipid-lowering treatment was more common in the MASLD-without-IBD group (58.8% vs. 36.8%), this difference was not statistically significant (*p* = 0.102). Total cholesterol levels did not differ significantly between the groups (*p* = 0.672). Similarly, no significant differences were observed in LDL cholesterol, HDL cholesterol, triglyceride levels or fasting plasma glucose levels between the groups.

### 3.4. Accuracy of CUN-BAE for Predicting the Presence of Liver Steatosis

**Figure 1:**
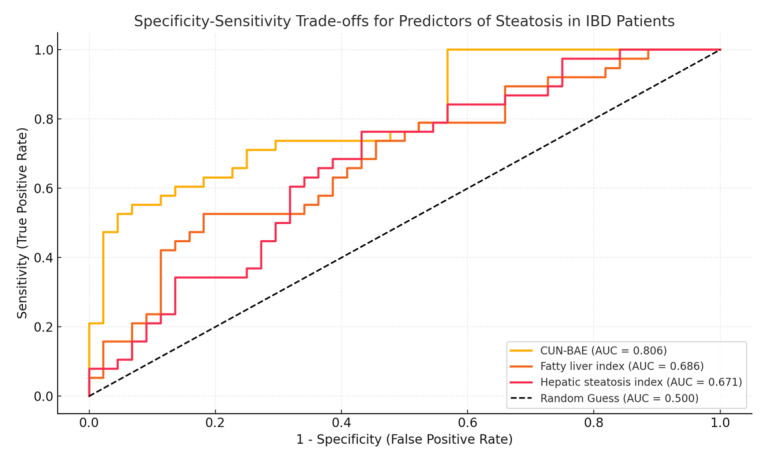
AUC for predictors of hepatic steatosis in IBD patients.

The diagnostic performance of various steatosis indices further illustrated differences between subgroups. The ROC curves revealed that the CUN-BAE index was the best predictor of liver steatosis, with an AUC of 0.806 in IBD patients. In contrast, traditional indices such as the FLI (AUC = 0.686) and HSI (AUC = 0.671) had lower predictive power in lean patients with IBD (Figure 1).

**Figure 2:**
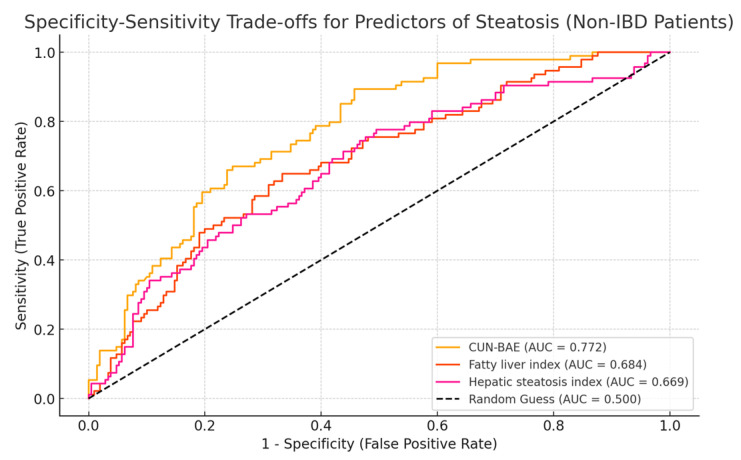
AUC for predictors of hepatic steatosis in non-IBD patients.

In non-IBD patients, as well, the AUROC curves for the predictors of steatosis demonstrated varying levels of discriminatory power, but followed the same trend as in IBD patients. The CUN-BAE index showed the highest ability to differentiate between patients with and without steatosis, as evidenced by its strong AUC, indicating excellent predictive accuracy, but slightly lower than in IBD patients. The FLI and HSI displayed moderate discriminatory performance, with an AUC slightly lower than that of CUN-BAE but still clinically significant (Figure 2).

### 3.5. Factors Associated with Liver Steatosis in Lean Patients

**Figure 3:**
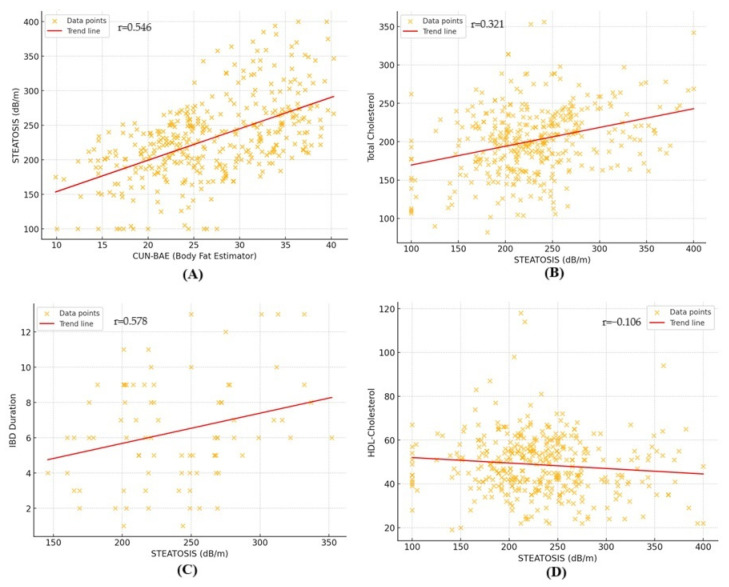
Correlation between CAP values and (**A**) CUN-BAE; (**B**) total cholesterol; (**C**) IBD duration in IBD patients; (**D**) HDL-cholesterol.

The correlation analysis aimed to investigate the association between CAP values and several clinical and biochemical parameters (Figure 3). The results revealed varying degrees of correlations across different variables. Lipid-related parameters, including total cholesterol (r = 0.321, r2 = 0.103) and triglycerides (r = 0.306, r2 = 0.093), presented weak but significant positive correlations. Similarly, indices related to body composition, such as CUN-BAE (r = 0.546, r2 = 0.298), showed a moderate association with steatosis. A weak negative correlation was observed for HDL cholesterol (r = −0.106, r2 = 0.011), suggesting an inverse relationship. The association between steatosis and fasting blood glucose levels was not statistically significant (r = 0.098, r2 = 0.009).

In the IBD cohort, a moderate positive correlation was observed between steatosis and IBD duration (r = 0.578, r2 = 0.334), indicating that patients with a longer disease duration tended to exhibit higher levels of hepatic steatosis. Moreover, the presence of pancolitis also showed a mild correlation with CAP values (r = 0.327, r2 = 0.106).

## 4. Discussion

The findings of this study contribute significantly to the growing understanding of MASLD, particularly in lean individuals, a subgroup that remains understudied despite its clinical significance. Moreover, the nomenclature change, as proposed by the Delphi consensus with the purpose of providing a more precise framework for better categorizing these patients, has also been evaluated [10].

The prevalence of liver steatosis is generally described to be lower in normal-weight patients when compared to non-lean patients. In our study, the prevalence of MASLD in lean subjects was 34.1%, which was slightly higher than reports from initial studies [27], but still in line with data from the current literature, which showed a prevalence between 25% and 50% [28].

Lean MASLD patients pose a paradox, as they often lack traditional markers of the metabolic syndrome, such as obesity, but still exhibit significant hepatic and cardiovascular morbidity. According to our findings, lean patients have significant metabolic dysfunction, such as increased fasting plasma glucose, dyslipidemia and abnormal liver enzymes, despite their normal BMI. These results are in line with the current literature, which found lean MASLD patients to have a distinct phenotype in which the lack of obesity does not provide protection against the progression of liver disease [29]. Moreover, although their BMI remained below the threshold for overweight, lean individuals with MASLD exhibited a higher BMI (23.3 ± 1.5 vs. 22.22 ± 1.83, *p* < 0.001) and WC (87.42 ± 3.99 vs. 84.64 ± 4.88, *p* < 0.001) compared to lean individuals without MASLD [12,30,31,32]. Additionally, visceral adiposity was more pronounced in lean individuals with MASLD than their non-MASLD counterparts, evaluated using CUN-BAE, in line with results from the current literature [30,33]. Triglyceride levels and HDL cholesterol levels showed a progressive change, with triglycerides increasing and HDL decreasing from lean individuals without MASLD to those with MASLD [30,31,32]. These findings suggest that visceral obesity, as indicated by elevated WC, may play a role in the development of MASLD in lean individuals. Furthermore, impaired lipid metabolism in these patients is evident from increased triglyceride levels and reduced HDL levels.

Recent research has shown that lean MASLD patients exhibit a comparable risk for hepatic fibrosis and steatosis, with some studies suggesting that they may even have a higher risk of liver-related mortality compared to their non-lean counterparts [34,35]. In our study, LSM values and the proportion of patients with significant liver fibrosis were considerably higher in the lean MASLD cohort (*p* < 0.001). On the other hand, there were no statistically significant differences regarding liver enzyme levels between the two groups, with slightly higher values in patients with MASLD.

In this study, despite significant clinical and metabolic differences between lean individuals with MASLD and their non-MASLD counterparts, key biochemical markers, including CRP, ALT, AST, GGT and ALP, did not differ significantly. This finding suggests that these parameters may have limited utility in distinguishing MASLD from non-MASLD individuals, particularly in lean patients. CRP, a widely used inflammatory marker, was not significantly elevated in MASLD patients compared to controls (*p* = 0.164), aligning with recent findings that highlighted the inconsistent association of CRP with liver inflammation in MASLD, particularly in non-obese individuals [36]. Similarly, ALT and AST levels, commonly used parameters in liver disease assessment, may remain within the normal range in MASLD, complicating early diagnosis. This is particularly relevant as long-term high-normal ALT levels have been proposed as a potential indicator of MASLD onset rather than an indicator of significant liver injury [37].

The biochemical profile of MASLD patients can be divided into two main categories, namely cholestatic and hepatocellular patterns. In our study, GGT and ALP levels also showed no significant differences between MASLD and non-MASLD individuals. Our findings are in line with the current literature, suggesting that in lean individuals with MASLD, these patterns may be less distinct, further complicating their use in clinical differentiation [38].

According to the relatively recent nomenclature change, MASLD is defined by the presence of at least one cardiometabolic risk factor in patients with liver steatosis. Regarding the presence of cardiometabolic risk factors defined by the Delphi consensus in MASLD patients, our cohort showed that 76.4% of the individuals had at least two cardiometabolic risk factors, high blood pressure being the most commonly found (39.3%). As a result, the presence of hepatic steatosis and fibrosis in lean patients, along with evidence of metabolic dysfunction, suggests that factors other than obesity, such as genetic predisposition, altered gut microbiota and low-grade chronic inflammation, could play a critical role in disease development [39].

The subgroup analysis of lean MASLD patients with IBD provides further evidence of the intricate interplay between the gut and liver health [40,41]. Lean IBD patients exhibited more severe hepatic steatosis and higher levels of systemic inflammation, as indicated by elevated CAP and CRP levels, respectively. These findings are consistent with the emerging research on the gut–liver axis, in which disruption of the intestinal barrier in IBD contributes to the pathogenesis of MASLD through increased intestinal permeability and subsequent hepatic inflammation. Chronic inflammation, as the hallmark of IBD, is characterized by elevated levels of pro-inflammatory cytokines that play a pivotal role in altering hepatic lipid metabolism [19,42]. The association between IBD and MASLD in lean individuals emphasizes the need for a multidisciplinary approach in managing these patients, incorporating both gastroenterological and hepatological expertise.

In our cohort, the prevalence of MASLD among IBD patients was significantly higher than in lean non-IBD patients (46.3% vs. 30.9%, *p* = 0.013). The association between the two pathologies is widely known and evaluated in the current literature, with IBD being recognized as an independent risk factor for MASLD in lean individuals [22,43]. Additionally, MASLD has been shown to significantly increase all-cause mortality risk in IBD patients, particularly in lean individuals [44]. Regarding IBD-related factors, disease activity, disease duration, prior surgery or extensive intestinal involvement have been associated with the development of hepatic steatosis [45,46]. In our cohort, a moderate positive correlation has been observed between steatosis and IBD duration, further emphasizing that the relationship between IBD and MASLD is complex and more than just a metabolic dysfunction.

This study highlights the superiority of CUN-BAE as a reliable tool for predicting liver steatosis, particularly in lean MASLD patients. While both HSI and FLI have been validated as practical tools for identifying hepatic steatosis in general populations, their performance in lean MASLD patients, particularly those with co-existing IBD, appears suboptimal. HSI and FLI both rely heavily on markers that can have normal values in lean MASLD individuals. As a result, these indices may underestimate the risk or presence of hepatic steatosis in this subgroup, leading to diagnostic inaccuracies. CUN-BAE integrates BMI with age- and sex-specific adjustments to estimate adiposity comprehensively, enabling the identification of steatosis in patients with normal BMI but abnormal fat distribution [16,25,26,47]. The superiority of CUN-BAE for investigating the metabolic-associated pathologies is recognized in the current literature. CUN-BAE performs better than the traditional indices like HSI and FLI in predicting MASLD, particularly in females, and has the capacity to predict cardiometabolic multimorbidity across populations [48,49].

Current guidelines recommend screening for MASLD using non-invasive tests in individuals with cardiometabolic risk factors, abnormal liver enzymes, or imagistic evidence of hepatic steatosis, especially those with type 2 diabetes or obesity, these being the metabolic diseases with the strongest impact on the natural history of MASLD. Management focuses on lifestyle interventions, including weight loss through diet and exercise, to improve liver health and reduce disease progression. In our study, even if the prevalence of hepatic steatosis was quite high, significant liver fibrosis was found in few patients, and mostly in those with more components of the metabolic syndrome. These findings suggest that screening for liver fibrosis in the general lean population would not be recommended, this being in line with current guidelines [24,50].

While this study provides important insights, it also has limitations that should be taken into account. The study was conducted in a single tertiary care center, which may limit the generalizability of the findings. Furthermore, the cross-sectional design prevents the establishment of causality between the observed relationships. Future research should focus on longitudinal studies to better understand the natural history of MASLD in lean individuals and the impact of targeted interventions on cardiovascular and hepatic outcomes. Another limitation is the reliance on the Fibroscan for the assessment of liver steatosis and fibrosis. While VCTE is a non-invasive and widely used method, it has its own limitations, such as variability in results due to operator dependency and patient factors. The use of indices such as CUN-BAE, FLI and HSI, while effective, may not fully capture the complexity of the disease, introducing potential biases in the estimation of clinical features. Additionally, the study did not include IBD-specific inflammatory markers, liver biopsy or molecular studies, limiting the ability to comprehensively characterize the underlying pathophysiology. The absence of serum cytokine measurements, such as IL-6, IL-1β and TNFα, further constrains the interpretation of systemic inflammatory responses. The circadian rhythm of the liver, which plays a significant role in metabolic and hepatic processes, was not taken into consideration. The lack of an evaluation of gut microbiota, an emerging factor in liver and metabolic diseases, also represents a missed opportunity to explore its potential contributions. The change in nomenclature from NAFLD to MASLD brings both positive and negative implications. On the positive side, the term MASLD offers a more accurate and patient-centered definition by focusing on the metabolic dysfunction at the core of the disease, such as obesity, insulin resistance and other related conditions. This shift helps to reduce the stigma previously associated with the terms “non-alcoholic” and “fatty”, which framed the disease by what it was not rather than by what it was. MASLD emphasizes a proactive focus on metabolic health and offers a more inclusive approach, allowing healthcare professionals to better target interventions and communicate the true drivers of the condition. However, the transition to a new term also comes with challenges. Medical professionals, patients and researchers must adapt to the new terminology, which may create some initial confusion and inconsistency in communication. Additionally, the need to update clinical guidelines and the research literature may take time, leading to a temporary lag in unified understanding. Despite these challenges, the change is ultimately a step toward a more precise and stigma-free characterization of liver health, centering the conversation around metabolic wellness.

## 5. Conclusions

This study highlights the unique clinical and metabolic characteristics of lean individuals with MASLD, a subgroup often overlooked in the broader context of metabolic liver disease. Furthermore, the higher prevalence of MASLD in IBD patients underscores the intricate interplay between the two pathologies and the role of the gut–liver axis’s role in disease occurrence and progression. The findings emphasize that MASLD can develop and progress in the absence of obesity, driven by factors such as visceral adiposity, dyslipidemia and chronic inflammation associated with IBD. Moreover, the study demonstrates the diagnostic superiority of the CUN-BAE index in detecting liver steatosis in lean individuals, especially those with IBD, compared to traditional indices like the FLI and HSI. These findings highlight the need for tailored diagnostic tools and multidisciplinary management strategies to address the unique metabolic and inflammatory profiles of lean MASLD patients. The clinical implications of this research call for an increased awareness of MASLD in lean individuals, particularly those with IBD, to enable early detection and intervention. Future studies should focus on the longitudinal progression of MASLD in this population and explore targeted therapeutic strategies to mitigate both hepatic and systemic complications.

## Figures and Tables

**Table 1:** Baseline characteristics of patients.

Parameter	Overall Cohort (Mean ± Std) *n* = 387	IBD Patients (Mean ± Std) *n* = 82	Non-IBD Patients (Mean ± Std) *n* = 305	*p*-Value
Age (years)	50.9 ± 15.52	40.68 ± 13.62	53.66 ± 14.85	<0.001
Female, *n* (%)	174 (44.9%)	46 (56.1%)	128 (41.5%)	0.064
Man, *n* (%)	213 (55.1%)	36 (43.9%)	177 (58.5%)	0.058
Height (cm)	169.13 ± 9.01	166.79 ± 10.06	169.77 ± 8.61	0.016
Weight (kg)	64.83 ± 8.97	64.07 ± 8.78	65.04 ± 9.02	0.378
BMI (kg/m^2^)	22.59 ± 1.79	22.94 ± 1.23	22.49 ± 1.91	0.011
Waist circumference (cm)	85.59 ± 4.77	85.770 ± 4.08	85.541 ± 4.94	0.669
MASLD, *n* (%)	132 (34.1%)	38 (46.3%)	94 (30.9%)	0.013
CAP value (dB/m)	230.29 ± 57.39	239.35 ± 47.13	227.84 ± 59.69	0.067
LSM (kPa)	6.22 ± 2.85	6.65 ± 1.9	6.11 ± 3.05	0.054
Platelet count (10^9^/L)	236.15 ± 75.69	252.26 ± 60.91	231.81 ± 78.73	0.013
CRP (mg/dL)	0.53 ± 2.31	0.72 ± 1.68	0.51 ± 1.22	0.033
Ferritin (mg/dL)	141.78 ± 98.36	153.57 ± 81.23	138.61 ± 102.37	0.165
Fasting plasma glucose (mg/dL)	101.96 ± 29.88	108.48 ± 25.57	100.20 ± 30.74	0.014
Urea (mg/dL)	36.77 ± 12.21	38.78 ± 12.15	36.23 ± 12.19	0.094
Creatinine (mg/dL)	0.82 ± 0.2	0.88 ± 0.5	0.83 ± 0.4	0.673
ALT (IU/L)	41.45 ± 39.41	42.28 ± 37.68	41.230 ± 39.92	0.825
AST (IU/L)	37.24 ± 31.24	32.79 ± 24.16	38.45 ± 32.83	0.085
GGT (IU/L)	69.54 ± 154.98	37.35 ± 25.09	48.23 ± 173.19	0.031
ALP (IU/L)	93.26 ± 50.14	84.74 ± 36.17	95.55 ± 53.11	0.033
Total cholesterol (mg/dL)	201.46 ± 43.72	214.31 ± 32.67	198 ± 45.68	<0.001
Triglycerides (mg/dL)	126.83 ± 64.19	145.65 ± 69.81	121.75 ± 61.73	0.006
LDL-Col (mg/dL)	128.78 ± 38.92	127.69 ± 38.98	129.07 ± 38.96	0.777
HDL-Col (mg/dL)	48.76 ± 13.45	43.92 ± 12.39	50.06 ± 13.45	<0.001
Lipid-lowering treatment, *n* (%)	53 (13.7%)	23 (28%)	30 (9.9%)	<0.001
Hypertension, *n* (%)	110 (28.5%)	20 (24.4%)	90 (29.6%)	0.425
T2DM, *n* (%)	59 (15.3%)	11 (13.4%)	48 (15.8%)	0.721
Active smoker, *n* (%)	86 (22.3%)	28 (34.1%)	58 (19.1%)	0.006
Previous smoker, *n* (%)	64 (16.6%)	21 (25.6%)	43 (14.1%)	0.021
CUN-BAE	26.84 ± 6.89	28.15 ± 6.61	26.48 ± 6.94	0.046
Fatty liver index	31.49 ± 18.25	31.41 ± 14.30	31.51 ± 19.20	0.956
Hepatic steatosis index	32.04 ± 4.25	34.52 ± 3.56	31.37 ± 4.17	<0.001

BMI, body mass index; MASLD, metabolic dysfunction-associated steatotic liver disease; CAP, controlled attenuation parameter; ALT, alanine aminotransferase; AST, aspartate aminotransferase; GGT, gamma-glutamyl transferase; ALP, alkaline phosphatase; LDL-Col, low-density lipoprotein cholesterol; HDL-Col, high-density lipoprotein cholesterol; LSM, liver stiffness measurement; CRP, c-reactive protein; T2DM, Type 2 diabetes; CUN-BAE, Clínica Universidad de Navarra-Body Adiposity Estimator.

**Table 2:** Comparison of MASLD vs. Non-MASLD lean patients.

Parameter	MASLD Patients (Mean ± SD) *n* = 132	Non-MASLD Patients (Mean ± SD) *n* = 255	*p*-Value
Age (years)	52.29 ± 14.67	50.19 ± 15.93	0.197
Height (cm)	169.75 ± 8.71	168.82 ± 9.17	0.329
Weight (km)	67.34 ± 8.51	63.54 ± 8.95	<0.001
BMI (kg/m^2^)	23.30 ± 1.50	22.22 ± 1.83	<0.001
Waist circumference (cm)	87.42 ± 3.99	84.64 ± 4.88	<0.001
CAP value (dB/m)	290.48 ± 40.30	199.01 ± 36.03	<0.001
LSM (kPa)	6.93 ± 2.39	5.86 ± 3.01	<0.001
Platelet count (10^9^/L)	232.23 ± 86.31	238.20 ± 69.64	0.493
Ferritin (mg/dL)	148.71 ± 114.77	138.19 ± 88.68	0.359
CRP (mg/dL)	0.97 ± 1.23	0.84 ± 1.46	0.164
Fasting plasma glucose (mg/dL)	107.94 ± 35.09	98.86 ± 26.33	0.014
Urea (mg/dL)	37.21 ± 10.60	36.54 ± 12.98	0.587
Creatinine (mg/dL)	0.88 ± 1.89	0.82 ± 2.15	0.443
ALT (IU/L)	45.17 ± 36.40	39.52 ± 40.82	0.166
AST (IU/L)	39.92 ± 36.50	35.86 ± 28.12	0.266
GGT (IU/L)	77.55 ± 159.16	65.39 ± 152.93	0.472
ALP (IU/L)	89.11 ± 42.59	95.42 ± 53.61	0.209
Total cholesterol (mg/dL)	216.48 ± 40.25	193.67 ± 43.49	0.018
Triglycerides (mg/dL)	150.91 ± 74.61	114.32 ± 54.12	<0.001
LDL-Col (mg/dL)	142.42 ± 35	121.69 ± 39.04	<0.001
HDL-Col (mg/dL)	46.42 ± 12.82	49.98 ± 13.65	0.012
Lipid-lowering treatment, *n* (%)	34 (25.7%)	19 (7.45%)	<0.001
Hypertension, *n* (%)	52 (39.3%)	58 (22.7%)	<0.001
T2DM, *n* (%)	30 (22.7%)	29 (11.3%)	<0.001
Active smoker, *n* (%)	42 (31.8%)	44 (17.2%)	0.014
Previous smoker, *n* (%)	26 (19.7%)	38 (14.9%)	0.094
CUN-BAE	31.21 ± 5.42	24.57 ± 6.49	<0.001
Fatty liver index	38.90 ± 18.03	27.65 ± 17.19	<0.001
Hepatic steatosis index	33.76 ± 4.39	31.15 ± 3.90	<0.001
Fibrosis stage, *n* (%)			
F0	55 (41.6%)	112 (43.9%)	
F1	41 (31.1%)	97 (38.1%)	
F2	27 (20.4%)	38 (14.9%)	
F3	7 (5.3%)	8 (3.1%)	
F4	2 (1.5%)	0	

BMI, body mass index; MASLD, metabolic dysfunction-associated steatotic liver disease; CAP, controlled attenuation parameter; ALT, alanine aminotransferase; AST, aspartate aminotransferase; GGT, gamma-glutamyl transferase; ALP, alkaline phosphatase; LDL-Col, low-density lipoprotein cholesterol; HDL-Col, high-density lipoprotein cholesterol; LSM, liver stiffness measurement; CRP, c-reactive protein; T2DM, Type 2 diabetes; CUN-BAE, Clínica Universidad de Navarra-Body Adiposity Estimator; F0: no fibrosis, F1: mild fibrosis, F2: significant fibrosis, F3: advanced fibrosis, F4: liver cirrhosis.

**Table 3:** Comparison of clinical profiles between IBD and Non-IBD patients with MASLD.

Parameter	MASLD and IBD Patients (Mean ± Std) *n* = 38	MASLD Patients Without IBD (Mean ± Std) *n* = 94	*p*-Value
Age (years)	47.18 ± 13.24	54.35 ± 14.78	0.008
Height (cm)	165.05 ± 9.66	171.64 ± 7.55	<0.001
Weight (kg)	64.73 ± 8.29	68.39 ± 8.41	0.025
BMI (kg/m^2^)	23.68 ± 0.97	23.14 ± 1.64	0.021
Waist circumference (cm)	88.43 ± 3.24	87.01 ± 4.21	0.038
CAP value (dB/m)	281.31 ± 28.45	273.19 ± 43.78	0.048
LSM (kPa)	6.94 ± 1.81	6.931 ± 2.58	0.977
Platelet count (10^9^/L)	247.92 ± 55.62	225.89 ± 95.51	0.101
Ferritin (mg/dL)	150.31 ± 77.73	148.06 ± 127.08	0.901
CRP (mg/dL)	0.93 ± 1.21	0.77 ± 1.54	0.043
Fasting plasma glucose (mg/dL)	106.97 ± 23.39	108.33 ± 38.94	0.806
Urea (mg/dL)	39.05 ± 12.12	36.46 ± 9.89	0.248
Creatinine (mg/dL)	1.02 ± 1.6	0.96 ± 2.48	0.195
ALT (IU/L)	38.68 ± 27.01	47.79 ± 39.39	0.130
AST (IU/L)	29.13 ± 16.27	44.27 ± 41.28	0.003
GGT (IU/L)	33.52 ± 17.41	95.34 ± 185.61	0.001
ALP (IU/L)	82.52 ± 37.17	91.77 ± 44.49	0.225
Total cholesterol (mg/dL)	218.52 ± 31.37	215.64 ± 43.45	0.672
Triglycerides (mg/dL)	155.15 ± 73.45	149.19 ± 75.38	0.676
LDL-Col (mg/dL)	142.94 ± 37.69	142.21 ± 34.06	0.916
HDL-Col (mg/dL)	43.61 ± 12.16	47.56 ± 12.96	0.101
Lipid-lowering treatment, *n* (%)	14 (36.8%)	20 (58.8%)	0.102
Hypertension, *n* (%)	16 (42.1%)	36 (38.4%)	0.724
T2DM, *n* (%)	9 (23.6%)	21 (22.3%)	0.892
Active smoker, *n* (%)	11 (28.9%)	31 (32.9%)	0.135
Previous smoker, *n* (%)	7 (18.4%)	19 (20.2%)	0.836
CUN-BAE	31.85 ± 4.96	30.94 ± 5.59	0.362
Fatty liver index	36.45 ± 14.26	39.88 ± 19.32	0.264
Hepatic steatosis index	35.65 ± 3.86	32.99 ± 4.37	<0.001
Fibrosis stage, *n* (%)			
F0	15 (39.5%)	30 (31.9%)	
F1	12 (31.5%)	39 (41.5%)	
F2	8 (21.1%)	19 (20.3%)	
F3	3 (7.9%)	4 (4.2%)	
F4	0	2 (2.1%)	

BMI, body mass index; CAP, controlled attenuation parameter; ALT, alanine aminotransferase; AST, aspartate aminotransferase; GGT, gamma-glutamyl transferase; ALP, alkaline phosphatase; LDL-Col, low-density lipoprotein cholesterol; HDL-Col, high-density lipoprotein cholesterol; LSM, liver stiffness measurement; CRP, c-reactive protein; T2DM, Type 2 diabetes; CUN-BAE, Clínica Universidad de Navarra-Body Adiposity Estimator; F0: no fibrosis, F1: mild fibrosis, F2: significant fibrosis, F3: advanced fibrosis, F4: liver cirrhosis.

## Data Availability

The data presented in this study are available on request from the corresponding author. The data are not publicly available because they are the property of the Institute of Gastroenterology and Hepatology, Iasi, Romania.
